# Saúde Digital – Ferramenta de Empoderamento e Igualdade de Gênero?

**DOI:** 10.36660/abc.20240739

**Published:** 2024-12-11

**Authors:** Larissa Espíndola, Alexandra Maria Monteiro Grisolia, Gláucia Maria Moraes de Oliveira

**Affiliations:** 1 Hospital Santa Izabel Salvador BA Brasil Hospital Santa Izabel, Salvador, BA – Brasil; 2 Universidade do Estado do Rio de Janeiro Rio de Janeiro RJ Brasil Universidade do Estado do Rio de Janeiro, Rio de Janeiro, RJ – Brasil; 3 Universidade Federal do Rio de Janeiro Rio de Janeiro RJ Brasil Universidade Federal do Rio de Janeiro, Rio de Janeiro, RJ – Brasil

**Keywords:** Tecnologias Digitais, Saúde Digital, FemTech

Nos últimos anos, o avanço das tecnologias digitais (TD) revolucionou diversos setores, e a saúde não é uma exceção. Ferramentas digitais como aplicativos de monitoramento, plataformas de telemedicina e até o uso de *wearables* permitem que indivíduos sejam protagonistas do seu autocuidado.^1^

Para as mulheres, essas inovações tecnológicas oferecem um potencial particularmente transformador, contribuindo para a superação de barreiras históricas no acesso aos cuidados de saúde, agora na palma da mão, e para promoção da sua saúde e prevenção do agravamento de doenças. O recente relatório da Organização Mundial da Saúde destaca que estas ferramentas podem melhorar as informações essenciais e o acesso a serviços vitais de saúde, e apoiar a saúde materna, promovendo assim a igualdade de gênero e a autonomia das mulheres na gestão da sua saúde, especialmente em regiões onde o acesso pode ser limitado devido a barreiras socioeconômicas ou culturais.^2^

A crescente digitalização da assistência médica apresenta uma oportunidade única para preencher essas lacunas, com o desenvolvimento de ferramentas digitais adaptadas às necessidades das mulheres, denominadas de "FemTech" (*female technology*). No entanto, embora tenham um potencial de preencher várias lacunas no que tange tanto os determinantes sociais em saúde, quanto a prevenção primária e secundária,^1^ melhorando a autogestão em saúde, encontramos barreiras significativas para sua implementação, especialmente pelos fatores socioeconômicos, e o desafio do letramento digital para a plena adoção da saúde digital (SD) com mobilidade.^2^

O mercado de FemTech mostrou crescimento significativo nos últimos anos. Em 2019, a indústria gerou aproximadamente 820 milhões de dólares em receita global e atraiu 592 milhões de dólares em investimentos. As projeções indicam que o mercado pode alcançar US$ 1,1 bilhão em 2024, refletindo um crescente interesse em atender as demandas femininas.^3^

No contexto da saúde feminina, as TD vão além de facilitar o acesso a serviços médicos. São ferramentas que oferecem oportunidades ímpares de empoderamento, bem como do estabelecimento de redes de apoio no cuidado e orientação em saúde, inclusive com outros países. Aumentam a autonomia das mulheres, melhorando sua capacidade de tomada de decisão, facilitando práticas de autocuidado e sistemas de automonitoramento personalizados, incluindo o acesso facilitado a programas de triagem, mudanças de estilo de vida e maior envolvimento no domínio de saúde pessoal. Assim, permitem que a mulher tome decisões mais conscientes sobre saúde, desde o planejamento familiar até a gestão de condições crônicas, passando pelo gerenciamento emocional e financeiro.^2^

A SD também tem o potencial de mitigar desigualdades regionais e socioeconômicas que historicamente impactam negativamente a saúde das mulheres. No Brasil, onde uma significativa parcela da população enfrenta dificuldades no acesso a cuidados de saúde, as ferramentas digitais se destacam como soluções eficazes e de relativo baixo custo pelo acesso por dispositivos móveis já utilizados em diferentes classes sociais. Exemplos disso são o atendimento médico ou multiprofissional remoto e oriundo de grandes instituições de ensino e serviço de excelência dentro do Sistema Único de Saúde (SUS) brasileiro, especialmente relevantes em áreas remotas ou marginalizadas, onde podem facilitar o acesso a cuidados de saúde essenciais, como consultas pré-natais e controle de doenças crônicas.^4^ Ademais, as ferramentas como a incorporação da Inteligência Artificial (IA) nos processos, os prontuários eletrônicos e o rastreio digital de dados de saúde permitem que os sistemas de saúde identifiquem padrões populacionais e implementem intervenções mais eficazes e direcionadas, com foco na equidade de gênero.

O uso de TD mostrou grande inovação na prevenção e diagnóstico precoce de doenças que afetam particularmente as mulheres, como o câncer de mama e de colo do útero. Ferramentas de IA e *Machine Learning* são capazes de analisar grandes volumes de dados para identificar sinais precoces de doenças, com uma precisão cada vez maior.^5^ Adicionalmente, os aplicativos de meditação, plataformas de suporte psicológico e ferramentas de *mindfulness* são cada vez mais acessíveis, ajudando mulheres a lidarem com o estresse, ansiedade, depressão e questões relacionadas ao bem-estar que impactam na saúde mental e cardiovascular.

No cenário de saúde do Brasil, as TD têm um papel crucial na expansão do acesso e na melhoria da qualidade da atenção primária à saúde da mulher. Se integradas aos serviços básicos de saúde, podem ajudar a superar desafios estruturais e promover a equidade no cuidado em diferentes especialidades médicas. Algumas evidências sugerem que as consultas mais direcionadas ao problema da mulher melhoram a promoção da saúde, a capacitação do paciente para o automonitoramento, a qualidade da manutenção de registro, além de ser um facilitador de diagnósticos mais precisos.^6^

Embora as TD ofereçam inúmeras oportunidades, a privacidade, a segurança e a confidencialidade dos dados das usuárias devem ser garantidas de acordo com a Lei Geral de Proteção de Dados, de forma a proteger informações sensíveis.^7-9^

Outro ponto a se considerar são as barreiras na implementação relacionadas à parte estrutural (a Estratégia de Governo Digital está em franca implementação e o Programa SUS Digital Brasil teve a adesão de 100% dos municípios), falta de capacitação profissional (letramento digital do profissionais e trabalhadores do SUS), falta de integração entre sistemas de saúde (em processo de implementação pela Rede Nacional de Dados em Saúde do SUS), custo operacional, e as barreiras relacionadas aos próprios pacientes (baixa educação em saúde, desigualdade socioeconômica, resistência cultural e social, falta de conhecimento claro sobre os benefícios da SD e letramento digital). Consolidar o processo de implementação como uma etapa sequencial e contínua da gestão de tecnologias é uma ação necessária para transpor essas barreiras.^10^

No horizonte da SD, tecnologias emergentes como a realidade aumentada e a realidade virtual têm potencial para mudar a forma como as mulheres interagem com os cuidados de saúde. Essas ferramentas podem ser usadas, por exemplo, para simulações de procedimentos médicos, treinamentos em saúde sexual e reprodutiva, ou até mesmo como suporte terapêutico em tratamentos de saúde mental.^1,2^

**Figura 1:**
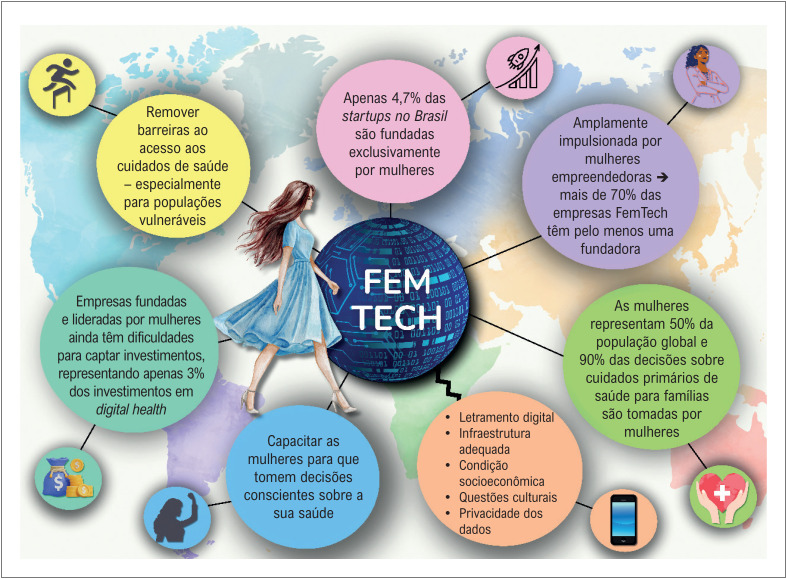
FemTech e seu impacto potencial na saúde da mulher.^12,13^

Desse modo, as TD apresentam um enorme potencial para revolucionar a saúde da mulher, promovendo acesso, equidade de gênero e empoderamento. A Figura 1 destaca o impacto potencial do mercado "FemTech", com suas vantagens e desafios. No entanto, para que as TD atinjam seu potencial pleno na saúde da mulher, é necessário coordenar esforços que envolvam academia, governo, instituições de saúde, profissionais e população em geral ^11^ para vencer as barreiras culturais, de infraestrutura e de educação digital, permitindo acesso a todas as mulheres, independente de sua localização geográfica ou condição socioeconômica.

**Figura 2:**
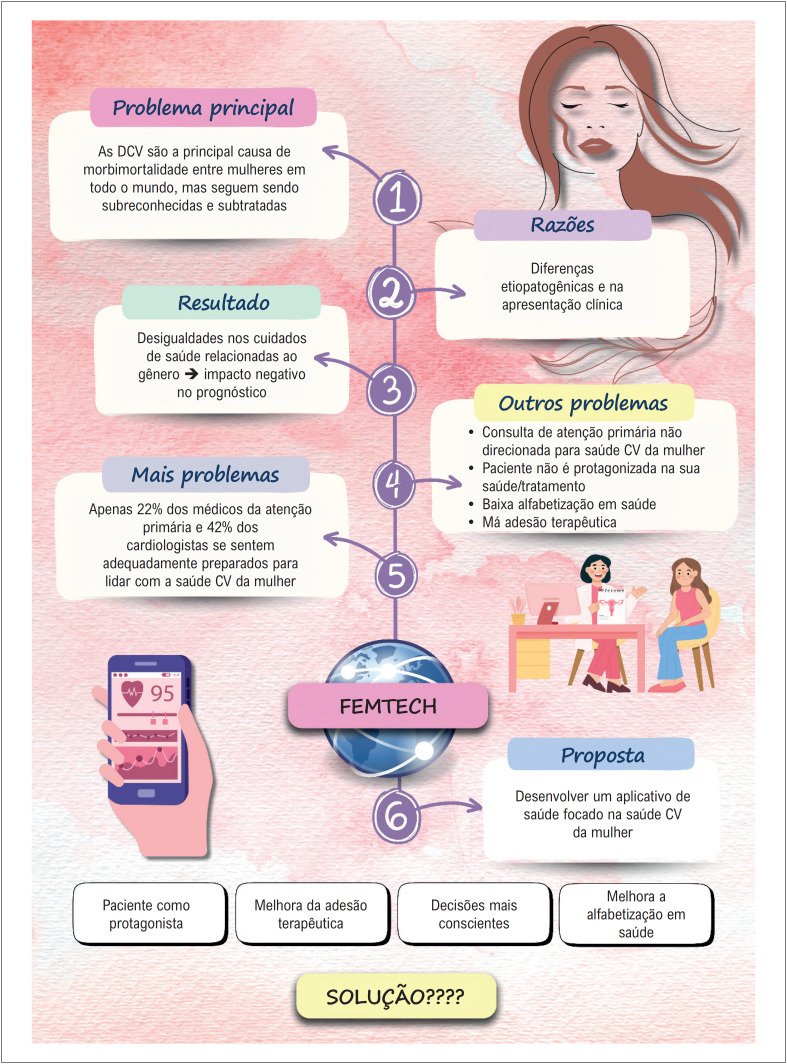
Proposta de solução digital para a saúde cardiovascular feminina no Brasil;^14^ CV: cardiovascular; DCV: doenças cardiovasculares.

Na Figura 2 mostramos os *gaps* relacionados à atenção primária no Brasil e propomos uma solução digital que pode impactar positivamente na saúde cardiovascular da mulher.

Acreditamos que o futuro da saúde da mulher é digital, e o Brasil tem potencial para liderar essa transformação.
