# Supplementary material for: Saúde Digital – Ferramenta de Empoderamento e Igualdade de Gênero?
Source: Arq Bras Cardiol. 2024 Dec 11;121(12):e20240739. [Article in Portuguese] doi: 10.36660/abc.20240739 (PMC11634308; doi:10.36660/abc.20240739)
Supplement: Translation

# Translation: Digital Health – A Tool of Empowerment and Gender Equality?

**DOI:** 10.36660/abc.20240739i

**Authors:** Larissa Espíndola, Alexandra Maria Monteiro Grisolia, Gláucia Maria Moraes de Oliveira

**Affiliations:** 1 Salvador BA Brazil Hospital Santa Izabel, Salvador, BA – Brazil; 2 Rio de Janeiro RJ Brazil Universidade do Estado do Rio de Janeiro, Rio de Janeiro, RJ – Brazil; 3 Rio de Janeiro RJ Brazil Universidade Federal do Rio de Janeiro, Rio de Janeiro, RJ – Brazil

**Keywords:** Digital Technology, Digital Health, FemTech

Over the last years, advances in digital technology (DT) have revolutionized various sectors, and health is not an exception. Digital tools including monitoring devices, telemedicine platforms and even wearables have enabled individuals to be responsible for their self-care.^1^

These technological innovations are particularly transformative for women, contributing to overcome a historical barrier to access to health care (now in the palm of their hands), and also to promote health and prevention of disease progression. The recent World Health Organization report highlights that digital health technologies can improve essential health information and the access to vital services, and support maternal health, thereby promoting gender equality, and women's autonomy in their care, especially in places where the access is inhibited by socioeconomical and cultural barriers.^2^

The increasing digitalization in health care represents a unique opportunity to fill those gaps, with the development of digital tools adapted to women's needs, the so called "FemTech" (female technology). However, despite their potential to solve issues related not only to social determinants in health but also to primary and secondary prevention,^1^ improving self-management in health, there are difficulties in their implementation, particularly due to socioeconomic issues and the challenge of digital illiteracy to the full adoption of digital health (DH) with mobility.^2^

The FemTech market has shown a significant increase over the past years. In 2019, the industry raised approximately 820 million dollars in global revenue and attracted 592 million dollars in investments. The market may reach 1.1 billion dollars in 2024, reflecting a growing interest in meeting women's needs.^3^

In the context of female health, DT not only facilitates the access to medical services, but also provides unique opportunities for empowerment, and establishment of support networks in health care and guidance, including with other countries. In addition, DT enhances women's autonomy, improving their decision-making capacity, and facilitating self-care practices and supporting personalized self-monitoring systems, including an easier access to screening programs, lifestyle changes and deeper involvement in personal health domains. Therefore, DT allows women to make more sensible decisions about health, from family-planning decisions, emotional and financial regulation, to management of chronic conditions.^2^

DT also has the potential to mitigate regional and socioeconomic disparities that have historically exerted a negative impact on women's health. In Brazil, where a significant proportion of the population faces difficulties in accessing healthcare services, digital tools have stood out as effective, relatively low-cost solutions, given the access through mobile devices, already a reality in different social classes. Examples include remote medical or multiprofessional assistance provided by large, renowned education and healthcare institutions included in the Brazilian Unified Health System (SUS). Remote care is particularly relevant in distant or marginalized areas, where access to essential health care, like prenatal care and control of chronic diseases is limited.^4^ Also, strategies like incorporation of artificial intelligence (AI) into processes, electronic medical records, and digital screening of health data, allow healthcare systems to identify population patterns and implement effective and targeted interventions focusing on gender equity.

The use of DT has been strikingly innovative in the prevention and early diagnosis of women's diseases like breast cancer and cervical cancer. AI and machine learning can analyze large volumes of data to identify initial signs of diseases, with higher accuracy.^5^ In addition, meditation apps, psychological support platforms and mindfulness tools have become more and more accessible, helping women to cope with stress, anxiety, depression, and well-being issues that affect both mental and cardiovascular health.

In Brazil, DT plays a crucial role in improving the access and the quality of primary care for women. If integrated into basic health services, DT may help overcome structural challenges and promote equity in the care provided in diverse medical specialties. Some evidence has suggested that women-centered care improves health promotion, patient empowerment in self-monitoring, the quality of record keeping, and diagnostic accuracy.^6^

Although DT offers numerous opportunities, patient privacy and safety, as well as data confidentiality should be ensured, following the General Data Protection Law, to protect sensitive information.^7-9^

One should also consider other barriers to DT implementation, including structure-related issues (the Brazilian "Digital Government" strategy has been implemented, and 100% of the cities have joined the Brazilian "SUS Digital" program), lack of professional capacity (poor digital literacy of healthcare professionals and SUS workers), lack of integration among health systems (in implementation process through the national health data system), operational costs, and patient-related barriers (poor health education, socioeconomic inequality, cultural and social resistance, lack of knowledge about the benefits of DH, and digital literacy. Consolidating the implementation process as a sequential and continuous step of technology management is crucial to overcome these barriers.^10^

In the context of DH, emerging technologies including augmented reality and virtual reality, can change the way women interact with healthcare practices. These tools can be used, for example, in medical procedure simulations, training in sexual and reproductive health, or even as therapeutical support in mental health treatment.^1,2^

Therefore, DT has a huge potential to revolutionize women's health, promoting access, gender equity and empowerment. Figure 1 highlights the potential impact of the "FemTech" market, with its advantages and challenges. However, to reach its full potential in women's health, coordinated efforts of the academy, government, healthcare institutions, professionals and population are needed^11^ to overcome cultural, infrastructure, and digital education barriers, allowing access to all women, regardless of their geographic location or socioeconomic status.

**Figure 1:** FemTech and its potential impact on women's health.^12,13^

Figure 2 illustrates the gaps related to primary attention in Brazil, and we propose a digital solution that can positively impact women's cardiovascular health.

**Figure 2:** Proposal of a digital solution for women cardiovascular (CV) health in Brazil;^14^ CVD: cardiovascular diseases.

We believe that the future of women's health is digital, and Brazil has the potential to lead this transformation.
